# Predominant Functional Expression of Kv1.3 by Activated Microglia of the Hippocampus after S*tatus epilepticus*

**DOI:** 10.1371/journal.pone.0006770

**Published:** 2009-08-26

**Authors:** Alexis Menteyne, Françoise Levavasseur, Etienne Audinat, Elena Avignone

**Affiliations:** 1 Institut National de la Santé et de la Recherche Médicale, Unité 603, Paris, France; 2 Université Paris Descartes, Paris, France; 3 Centre National de la Recherche Scientifique, Unité Mixte de Recherche 8154, Paris, France; Tokyo Medical and Dental University, Japan

## Abstract

**Background:**

Growing evidence indicates that the functional state of microglial cells differs according to the pathological conditions that trigger their activation. In particular, activated microglial cells can express sets of Kv subunits which sustain delayed rectifying potassium currents (Kdr) and modulate differently microglia proliferation and ability to release mediators. We recently reported that hippocampal microglia is in a particular activation state after a *status epilepticus* (SE) and the present study aimed at identifying which of the Kv channels are functionally expressed by microglia in this model.

**Methodology/Principal Findings:**

SE was induced by systemic injection of kainate in CX3CR1^eGFP/+^ mice and whole cell recordings of fluorescent microglia were performed in acute hippocampal slices prepared 48 h after SE. Microglia expressed Kdr currents which were characterized by a potential of half-maximal activation near −25 mV, prominent steady-state and cumulative inactivations. Kdr currents were almost abolished by the broad spectrum antagonist 4-Aminopyridine (1 mM). In contrast, tetraethylammonium (TEA) at a concentration of 1 mM, known to block Kv3.1, Kv1.1 and 1.2 subunits, only weakly reduced Kdr currents. However, at a concentration of 5 mM which should also affect Kv1.3 and 1.6, TEA inhibited about 30% of the Kdr conductance. Alpha-dendrotoxin, which selectively inhibits Kv1.1, 1.2 and 1.6, reduced only weakly Kdr currents, indicating that channels formed by homomeric assemblies of these subunits are not important contributors of Kdr currents. Finally, agitoxin-2 and margatoxin strongly inhibited the current.

**Conclusions/Significance:**

These results indicate that Kv1.3 containing channels predominantly determined Kdr currents in activated microglia after SE.

## Introduction

Recent experimental evidence has considerably expanded our knowledge of the biology and functions of microglia, the brain resident macrophages. First, it is now acknowledged that microglial cells in the healthy brain is not in a resting or dormant state but rather have an active surveying function, constantly exploring the cerebral parenchyma [1], [2]. Second, the activation state developed by microglia in response to various stimuli is not unique: different stimuli and different contexts lead microglia to develop different functional states which correspond to a diversity of functions of microglia underlying their deleterious or beneficial effects on neuronal survival and function (for review, [3]. Third, new roles for microglia have also been proposed in non-pathological contexts. In particular, microglia regulates neuronal death which occurs normally during brain development [4], as well as synaptogenesis (for review, [5]).

Potassium channels play a pivotal role in the activation process of microglia. Surveying non-activated microglia expresses little if any voltage-activated potassium channels (Kv) whereas large inward rectifying (Kir) and delayed rectifying outward potassium (Kdr) currents have been observed in activated microglia (for review, [6]). Interestingly, the expression pattern of these two types of potassium currents varies upon experimental and activation conditions [7]–[14]. Furthermore, as far as Kdr channels are concerned, several subunits have been identified in microglia and seem to control different functional aspects of its activation. Indeed, both Kv1.3 and Kv1.5 channels modulate the proliferation of activated microglial cells [13], [15]. In addition, Kv1.3 channel blockers reduce the NADPH-mediated respiratory burst of activated microglia and therefore the subsequent production of superoxide and free radicals which contributes to the deleterious effect of these cells on neuronal survival [16]. A role for Kv1.5 channels in controlling the LPS-induced release of nitric oxide by microglia has been proposed [13], although a calcium-activated potassium channel, KCa3.1, modulates also this release [17]. Finally, the expression of Kv1.1, Kv1.2 and Kv3.1 channels is up-regulated in some models of microglia activation and could regulate the production of pro-inflammatory signalling molecules such as IL-1beta, IL-6, TNFalpha and nitric oxide [18]–[20].

We have recently shown that microglial cells are in a particular activation state 24 to 48 h after a *status epilepticus* (SE). From a functional point of view, this state is characterized by an up-regulation of all their responses mediated by the activation of ATP receptors and the expression both Kir and Kdr currents [10]. Because the identity and the level of expression of the Kv channels mediating Kdr currents is finely tuned according to the activation state and determines other parameters of microglia activation (see above), the present study aimed at identifying which of the Kv channels are functionally expressed by microglia after SE. We induced SE by systemic injection of kainate in CX3CR1^eGFP/+^ mice in which microglia expresses the green fluorescent protein [21] and performed electrophysiological and pharmacological analyses of Kdr currents of microglia in acute hippocampal slices prepared 48 h after SE, i.e. at the peak of functional activation of microglia in this model [10]. Our results provide strong evidence for the predominant functional expression of Kv1.3 channels in microglia activated by a SE.

## Materials and Methods

### Animals and seizure induction

All experiments followed Inserm and European Union and institutional guidelines for the care and use of laboratory animals (Council directive 86/609EEC). The heterozygous CX3CR1^eGFP/+^ mice used throughout this study were obtained by crossing CX3CR1^eGFP/eGFP^ [21] with C57BL/6 (Janvier, Le Genest Saint Isle, France) wild type mice. Intra-peritoneal (i.p.) injection of kainate (18–22 mg/kg) in phosphate buffer saline (PBS) was used to induce a *status epilepticus* in 30 to 40 day-old mice, and age matched PBS injected animals were used as control. Animals were observed and classified according to the Racine scale: 1) freezing behaviour; 2) rigid posture with straight and rigid tail; 3) repetitive head bobbing, rear into a sitting position with forepaws shaking; 4) rearing and falling, jumping, running with period of total stillness; 5) continuous level 4; 6) lost of posture and generalized convulsion activity, usually preceding death. After kainate injection, mice showed progression through the different stages, usually entering in phase 1 about 15 minutes after the injection and reaching stage 3 in 30–45 minutes. Animals not showing the normal progression were re-injected with half dose. Only animals reaching at least stage 4 were considered for this study. The duration of crises varied from 2 to 4 hours and mortality was around 20%.

### Hippocampal slice preparation and electrophysiological recordings

Hippocampal slices were prepared 48 h after the induction of SE or after the i.p. injection of PBS for control animals. Mice were killed by cervical dislocation, the brain was then quickly removed and placed in ice-cold artifical cerebrospinal fluid (aCSF) bubbled with carbogene (95% O_2_/5% CO_2_) and in which NaCl was replaced with sucrose (in mM: 210 sucrose, 2.5 KCl, 26 NaHCO_3_, 1.25 NaH_2_PO_4_, 25 glucose, 1 CaCl_2_, 7 MgSO_4_; pH 7.4, osmolarity ∼310 mOsm). Transverse 350 µm thick slices were cut using a vibratome, transferred to a heated (34°C) holding chamber containing oxygenated (95% O_2_/5% CO_2_) standard aCSF (in mM: 124 NaCl, 3 KCl, 26 NaHCO_3_, 1.25 NaH_2_PO_4_, 10 glucose, 2 CaCl_2_, 1 MgCl) for 1 h, and then subsequently maintained at room temperature.

Individual slices were transferred to a recording chamber on the stage of an Olympus microscope (BX50WI) with a 40x water immersion onjective, equipped with cell-R imaging station including MT20 illumination system (Olympus, France) and a CCD camera (Hamamatsu ORCA2-AG, France). Slices were constantly perfused at room temperature (21–24°C) with oxygenated aCSF (3 ml/min). All drugs were bath applied. Visually-identified eGFP-expressing microglial cells located at least 30 µm below the slice surface were patched in whole-cell configuration in the *stratum radiatum* of the CA1 region of hippocampus. Micropipettes (5 to 7 MΩ) were filled with a solution containing (in mM): K-gluconate 132, HEPES 11, EGTA 0.1, MgCl_2_ 4 (pH 7.35 adjusted with KOH, osmolarity ∼300 mOsm). All potential values given in the text for experiments performed with this solution were corrected for a junction potential of 10 mV. For measuring Ca^2+^-activated potassium currents the intra-pipette solution contained (in mM) KCl 120, BAPTA 5, MgCl_2_ 2, Hepes 10 with 0 or 4.43, CaCl_2_.

Voltage-clamp recordings were performed using an Axopatch 200B (Molecular Devices, Sunnyvale, CA, USA). Currents were low-pass filtered at 5 kHz, collected using PClamp 9 (Molecular Devices, Sunnyvale, CA, USA) at a frequency 10 kHz and analyzed off line using Clampfit (Molecular Devices, Sunnyvale, CA, USA), ORIGIN (7.5, Origin Lab corporation, MA, USA), and custom made program in MATLAB. An electrophysiological characterization in voltage-clamp was made at the beginning of the recording. Hyperpolarizing and depolarizing steps (from –150 to +30 mV for 50 ms) were used to determine I/V relationship of each recorded cell. Membrane input resistance and capacitance of the cells were determined from the current responses to voltage pulses ranging from – 20 mV to +20 mV from a holding potential of −70 mV. As previously described [10], the I/V relationship of microglia from control animal was always linear whereas that of microglia from epileptic animals showed inward and outward rectifications. However, there was some variability in the amplitude of the currents underlying these rectifications in activated microglia [10]. For the purpose of the present study we thus selected activated microglial cells which responded to a step from −70 to +30 mV by an outward current of at least 50 pA after leak subtraction (see below).

Activation and inactivation curves of the outward currents activated by depolarization were obtained using a single protocol: starting from a holding potential of −70 mV, a series of 1 s duration pre-pulses ranging from −90 to +50 mV were applied, followed by a 200 ms pulse test at +50 mV. The activation and inactivation curves were obtained measuring the peak of the current in the pre-pulse and pulse test respectively. Then the leak current was subtracted. The leak current was determined by fitting the linear part of the I/V curve (from −80 to −50 mV). Conductance was calculated, normalized to its maximal value and plotted against holding or pre-pulse potential. The curve were fitted with the Boltzmann equation G/G_max_ = 100/(1+exp^((V^_1/2_^-V)/k)^), where V_1/2_ is the voltage at which the current is half activated, and k is the slope factor of the activation curve. The inactivation time constant was obtained by fitting the decay of the current evoked by a 1s pulse from −70 to +30 with a monoexponential curve. To determine the cumulative inactivation a series of 10 pulses of 200 ms of duration was applied at different frequencies, and after leak subtraction, currents were normalized to the first pulse.

The effects of drugs on potassium currents was assessed by acquiring I/V curves (from −150 to +50) with 5 sec intervals between different pulses in control and after 10 minutes of drug application. Leak current were subtracted off line, conductances were determined and normalized to the maximal conductance. Statistical significance was tested with paired t-test on currents at +30 mV after leak subtraction. Temporal matched experiments were performed without drug application to exclude variation of the delayed rectifying current under study. Data values are presented as mean±SEM. Statistical significance was tested with the program GraphPAd Instat (GraphPad Instat 3.06). Statistical significance was established at *p<0.05 and **p<0.01.

### Reagents

Alpha-dendrotoxin, recombinant agitoxin-2 and recombinant margatoxin were purchased from Alomone labs (Jerusalem, Israel). In experiments involving toxins, 0.1% bovine serum albumin was added to the extracellular solution to limit binding of the toxin to tubing and chamber. 4-Aminopyridine (4-AP), was purchased from Tocris Bioscience (Bristol, UK), kainate from Ascent Scientific (Weston-Super-Mare, UK); tetraethylammonium (TEA) and other chemicals were purchased from Sigma-Aldrich (Lyon, France).

## Results

**Figure 1:**
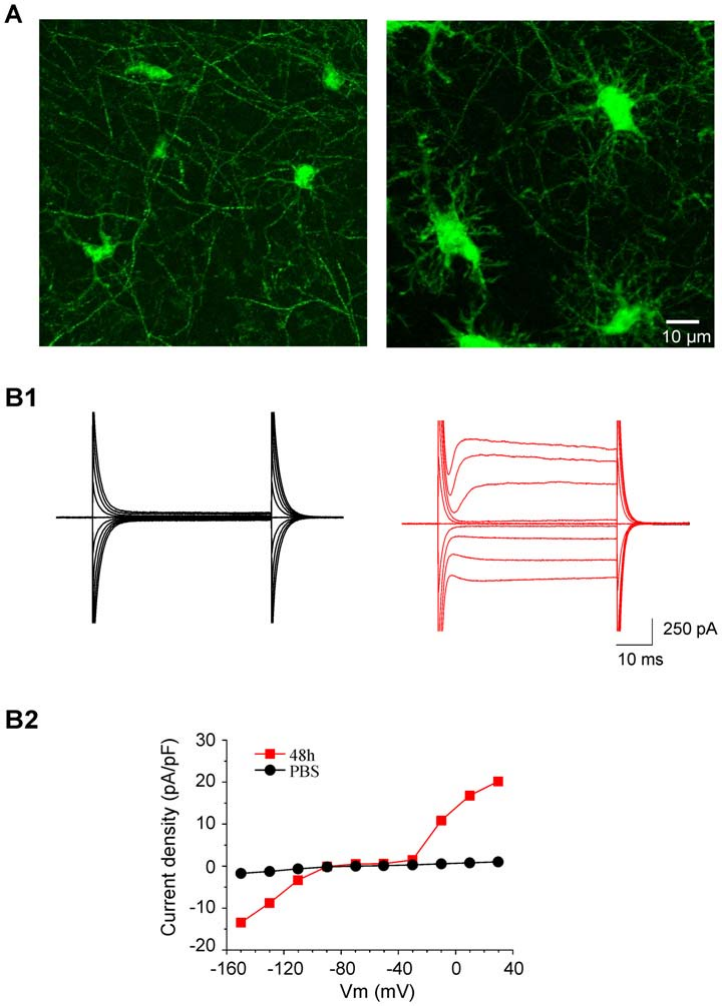
Activation of microglial cells 48 h after *status epilepticus* (SE) induced by intra-peritoneal injection of kainate. A Confocal images of fluorescent microglial cells in the stratum radiatum of the CA1 region of the hippocampus of CX3CR1^egfp/+^ mice in control conditions (left) or 48 h after SE (right). Each image is a maximum intensity z-projection of 31 confocal slices for a total thickness of 12 µm. B1 Examples of current responses induced by voltage steps of 20 mV increment from −150 to +30 mV (holding potential −70 mV) in microglial cells in control (left, black traces) and 48 hours after the induction of *status epilepticus* (right, red traces). B2 I/V curves of the cells showed in B1 normalized to their capacitance. The activated microglial cell expressed both inward and outward rectifying currents (red plot).

*Status epilepticus* (SE) was induced by an intra-peritoneal injection of kainate (see materials and methods) in CX3CR1^egfp/+^ mice. As previously described [10], the activation of hippocampal microglia gradually evolved during the first days that follow SE. The maximum of this activation process peaks around 48 h after SE and at this stage microglial cells have acquired a larger soma with thicker primary processes than resting or surveying microglia (Fig. 1A). Whole-cell recordings performed in acute hippocampal slices showed that the current-voltage relationship of microglia recorded in the stratum radiatum of the CA1 region of control mice was almost linear between membrane potentials of −140 to +40 mV (Fig. 1B). In contrast, the I/V curve of activated microglia recorded after SE was characterized by marked rectifications at hyperpolarized and depolarized membrane potentials (Fig. 1B; see also Materials and Methods for sampling of activated microglia). As described in the introduction, several potassium channels can potentially be responsible for the outward currents evoked by depolarizing voltage steps and we therefore set up to study the biophysical and the pharmacological properties of these currents.

### Biophysical properties of Kdr channels in activated microglia

**Figure 2:**
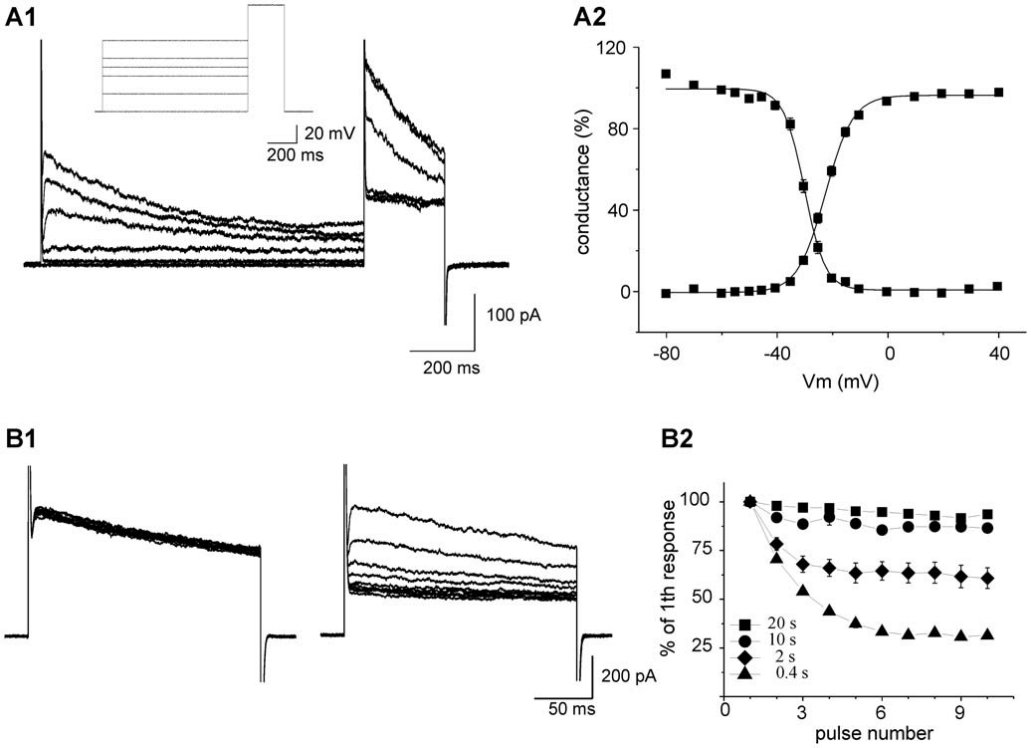
Biophysical properties of potassium channels expressed in microglial cells after *status epilepticus*. A1 Example of currents used to obtain activation and inactivation curves in microglia after *status epilepticus*. Microglia cells were held at a potential of −70 mV and stepped at different potentials for 1 s before a final step of 250 ms at +50 mV. The inset represents the voltage steps corresponding to the current traces showed in the figure. A2 Average of activation and inactivation curves (n = 13) normalized to maximal conductance. B1 Examples of currents induced in another microglial cell by 10 consecutive pulses from −70 to +30 mV with 30 s interval (left panel) or 0.4 s interval (right panel). B2 Evolution of the peak current amplitude obtained at every pulse expressed as percentage of the first response for 7 tested cells. Note that the smaller is the inter-pulse interval, the higher is the inactivation of the current.

We first characterized the biophysical properties of the channels activated by depolarization to compare them with those reported for other models of microglia activation. Activation and steady-state inactivation curves were constructed from normalized currents generated during the protocol shown in the inset of Figure 2A1 and were fitted to Boltzmann equations (see also Materials and Methods). Outward currents had an activation threshold near −35 mV and were fully activated above 0 mV. They were characterized by a half-maximal activation potential (V_1/2_) of −22.6±0.5 mV (with a slope factor of 4.9±0.2 mV; n = 13) and a steady-state half inactivation potential of –30.2±0.52 mV (with a slope factor of 3.6±0.3 mV n = 13; Fig. 2A2). During a long duration pulse at +40 mV, the current inactivated with a time constant of 294±12 ms (n = 24). Beside their steady-state activation and inactivation characteristics, potassium channels formed by different Kv subunits can also differ by the magnitude of their cumulative inactivation in response to repetitive depolarizing pulses applied with short inter-pulse intervals (see for instance [22]). We therefore studied cumulative inactivation of the outward currents evoked in activated microglia in responses to a series of 10 depolarizing 200 ms steps at +30 mV (from a holding of −70 mV) applied every 400 ms; 2 s, 10 s or 20 s. As shown in figure 2B, the amplitude of the outward current remained stable (or only slightly decreased) during the 10 pulses at low frequencies (below 0.1 Hz) but gradually decreased during the first pulses evoked with frequencies of 0.5 Hz and 2.5 Hz. In the latter case, the outward current amplitude reached a steady state at the 7^th^ pulse which amounted approximately 30% of its initial value. Altogether, the biophysical properties of Kdr currents of activated microglia resemble those of channels made up of Kv1.3 rather than of the other Kv subunits, in particular Kv1.5 or Kv3.1 which activate at more depolarized potentials and show limited steady-state and cumulative inactivation.

### Pharmacological properties of Kdr channels in activated microglia

**Figure 3:**
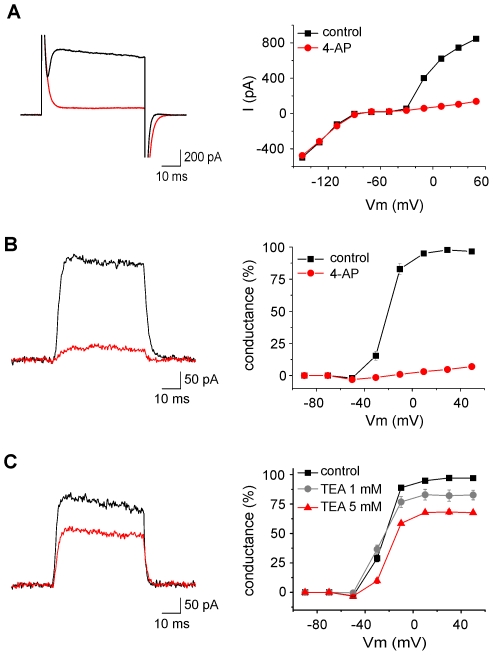
Effects of broad spectrum blockers of potassium channels on the outward rectifying current expressed by microglia 48 h after the induction of *status epilepticus*. A Example of the current (left panel) induced by a voltage step from −70 to +30 mV in control (black trace) and after perfusion of 4-AP (1 mM, red trace), and the I/V relationships in the same cell (right panel). Note that 4-AP almost completely abolished the outward rectifying currents without affecting the inward currents. B–C Examples of leak subtracted currents induced by a voltage step from −70 to +40 mV in control (black trace) and after 4-AP 1 mM (B, red trace) or TEA 5 mM (C). The leak conductances of the cells in B and C were 585 and 256 pS, respecitively. The graphs on the right represent the conductance, normalized to its maximum value, as a function of membrane potential and its inhibition induced by 4-AP (B, n = 8), TEA (C) 1 mM (n = 9) and 5 mM (n = 7).

**Figure 4:**
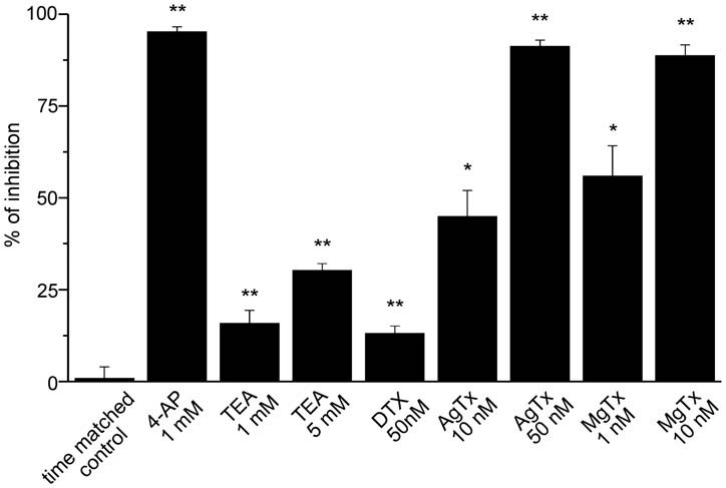
Summary of the effects induced by the different drugs tested on the outward rectifying potassium current evoked by a voltage step from −70 to +30 mV. The histogram represents the average of the leak subtracted current after drug application and normalized to its pre-drug value. The “time matched control” bar corresponds to experiments in which the current was measured during 10 to 15 minutes without any drug application to control for the absence of any significant run-down of the current. Statistical tests were done on raw data (paired *t test*, **p<0.01; the number of tested cells for each condition is that given in the legends of figures 3 and 4).

Consistent with the activation of Kdr channels, the outward currents were fully blocked by intracellular caesium (not shown) and by extracellular 4-AP. As shown in figure 3A, bath application of 1 mM 4-AP had no effect on the inward rectification or on the linear portion of the I/V curve but largely decreased the outward currents. Figure 3B shows the dramatic effect of 4-AP on the normalized conductance (see material and methods) of Kdr currents plotted as a function of the membrane potential. On average, 4-AP blocked 94.5±1.3% (n = 8, p<0.01, paired t test) of the outward current (leak-subtracted) induced by a step at +30 mV (Fig. 4). This concentration of 4-AP should block all Kv subunits identified so far in microglia, except Kv1.6 which should be only partially inhibited (see table 1).

**Table 1:** Biophysical and Pharmacological properties of cloned Kv-containing channels potentially expressed in microglia.

Biophysical properties	Kv 1.1	Kv 1.2	Kv1.3	Kv1.5	Kv1.3/1.5 heteromer	Kv1.6	Kv3.1	Present study
*Activation V1/2 mV)*	−32 [24]	+27 [24]	−23 [25]	−6.7 [25]	−20 [25]	−17 [27]	16 [24]	−22.6±0.5
	−29 [39]	−34 [39]	−26 [24]	−3 to −25 [24]	−7.2 to 6.3 [22]	−11 [29]	12.5−16.9 [31]	
	−26 [28]		−13 to −25 [32]	−19 [33]				
			−26 to −35 [40]	−11 [34]				
				0 to −15 [35]				
			−25 [39]	−8.6 [30]				
*Steady-state*	−47 [39]	−44 [39]	−44 [39]	−33 [33]			−9.6 [31]	–30.2±.52
*inactivation*	−45 [28]			−10 to −33 [35]			−17 [30]	
*V1/2 (mV)*				−38 to −41 [37]				
				−26 [30]				
*Inactivation*			526 [25]	1,300; 17,000 [33]	833–1470 [25]			294±12
*time constant*				5000 [35]	365–1740 [22]			
*(msec)*								
				>5000 [25]				
*Cumulative inactivation*	No [24]	No [24]	Yes [24]	No [24]		No [29]	No [24]	Yes
**Pharmacological Properties (IC 50)**								
*4 AP*	290 µM [24]	590 µM [24]	195 µM [24]	270 µM [24]		1.5 mM [27]	29 µM [24]	
	200 µM [28]		200–1500 mM [32]	0.1–400 mM [32]		0.3 mM [29]		
			1500 mM [41]					
*Alpha – DTX*	20 nM [24]	17 nM [24]	250 nM [24]	>1 µM [24]		20 nM [27]	>1 µM [24]	
	100 nM [28]					25 nM [29]		
*TEA*	0,3 mM [24]	560 mM [24]	10 mM [24]	330 mM [24]		7 mM [27]	0.2 mM [24]	
	0.4 mM [28]		11–50 mM [32]	40–330 mM [32]		1.7 mM [29]		
			10–11 mM [40]					
*AgTx 2*	44 pM [37]		4 pM [37]			37 pM [37]		
*MgTX*	30 pM [25]		30 pM [36]	>1 µM [25]		5 nM [40]	>200 nM [36]	

Numbers in brackets correspond to the articles referenced in the reference list.

We then tested the effect of TEA, another large spectrum inhibitor of potassium channels. At a concentration of 1 mM, TEA should block mostly Kv1.1 and Kv3.1 but not Kv1.2, Kv1.3 and Kv1.5 (see table 1). As shown in figure 3C, bath application of 1 mM TEA inhibited only slightly the outward current evoked in activated microglia. However, increasing TEA concentration to 5 mM, which should also partially block Kv1.3, decreased more significantly the currents (Fig. 3C). Comparison of the activation and inactivation curves obtained from the same cells before and after application of 5 mM TEA did not reveal any significant shift of the potentials of half-maximal activation or inactivation (n = 7, p = 0.83 and p = 0.89, respectively, paired t test). Such shifts could have been expected if Kv3.1, which activate and inactivate at more depolarized values, would have contributed substantially to the current. On average, an inhibition of 15.8±3.6% (n = 9, p<0.01, paired t test) and 30.2±1.2% (n = 7, p<0.01, paired t test) of the current evoked by a step at +30 mV was observed with 1 mM and 5 mM TEA, respectively (Fig. 4). Experiments in absence of any blocker and matching the temporal course of drug testing experiments did not reveal any significant change (Fig. 4, p = 0.97, paired t test, n = 5). These results suggest that Kv1.1 and Kv3.1 have not a major contribution to the microglia outward currents.

**Figure 5:**
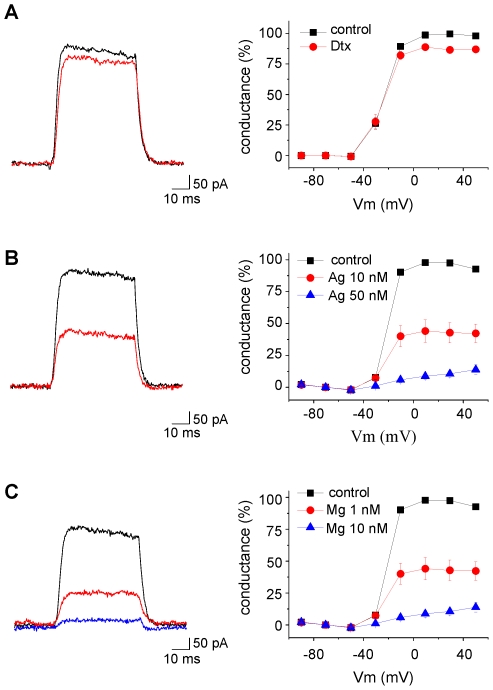
Effects of α-dendrotoxin (A, 50 nM), agitoxin-2 (B, 10 and 50 nM) and margatoxin (C, 1 and 10 nM) on the leak subtracted current induced by a voltage step from −70 to +40 mV (black traces recorded in control, red traces after drug application). The leak conductances of the cells in A, B and C were 338, 862 and 332 pS, respectively. The graphs on the right represent the conductance, normalized to its maximum value, as a function of the membrane potential and its inhibition by α-dendrotoxin (A, n = 4, Dtx), agitoxin-2 (B, AgTx, n = 14 for 10 nM, n = 6 for 50 nM) and margatoxin (C, MgTX, n = 8 for 1 nM, n = 11 for 10 nM).

We then tested if the snake toxin α-dendrotoxin which blocks Kv1.1, Kv1.2 and Kv1.6 with nanomolar affinity (see table 1) had any major effect on microglia outward currents. Bath application of 50 nM α-dendrotoxin slightly affected the delayed rectified current (Fig. 5A). On average the current evoked by a step at +30 mV was inhibited by 13±2% (Fig. 4, n = 4 p<0.001, paired t test), suggesting that Kv1.1, Kv1.2 and Kv1.6 have only a minor contribution.

Thus, the effect of 4-AP which virtually abolishes all outward currents is probably due to a blocking of Kv1.3 or Kv1.5 containing channels. Unfortunately, there is no specific blocker of Kv1.5 subunits. Lagrutta et al. (2006) showed that DPO-1 blocks homomeric Kv1.5 channels with nanomolar affinities but according to the same authors, this drug showed little or no selectivity for Kv1.5 over other Kv1.x mediated currents [23]. We therefore tested the effect of recombinant agitoxin-2 (AgTx) which potently blocks homomeric Kv1.3 channels at low nanomolar concentrations [15], [24]. As shown on figure 5B, the Kdr conductance was partially inhibited upon application of 10 nM AgTx and was almost completely blocked with a concentration of 50 nM. On average, 10 nM and 50 nM of the toxin inhibited the outward currents induced by a step at +30 mV by 44.9±7.1% (n = 14 p<0.01, paired t test) and 91.1±1.8% (n = 6 p<0.05, paired t test), respectively (Fig. 4). The fact that high concentrations of AgTx were needed to block microglial Kdr currents was somehow surprising given the high affinity of the toxin for Kv1.3 (or even for Kv1.1 and Kv1.6; see table 1). A lower accessibility of the toxin in acute slices or a possible lower affinity of the toxin for heteromeric combinations containing Kv1.3 could explain this result. We therefore tested another toxin, margatoxin, which is known to block Kv1.3 homomers and Kv1.3/Kv1.5 heteromers with different affinities [25]. We tested two different concentrations of margatoxin: 1 nM which partially blocks Kv1.3 homomers but had no effect on Kv1.3/Kv1.5 heteromers and 10 nM which fully blocks Kv1.3 homomers and only partially Kv1.3/Kv1.5 heteromers [25]. As shown on figure 5C, 1 nM margatoxin blocked already substantially Kdr currents which were abolished by a concentration of 10 nM. On average, 1 nM and 10 nM of the toxin inhibited the outward currents induced by a step at +30 mV by 55.9±8.3% (n = 7 p<0.05, paired t test) and 88.7±2.9% (n = 11 p<0.01, paired t test), respectively (Fig. 4). Thus, the effects of margatoxin rather support a predominant expression of homomeric Kv1.3 channels in activated hippocampal microglia.

**Figure 6:**
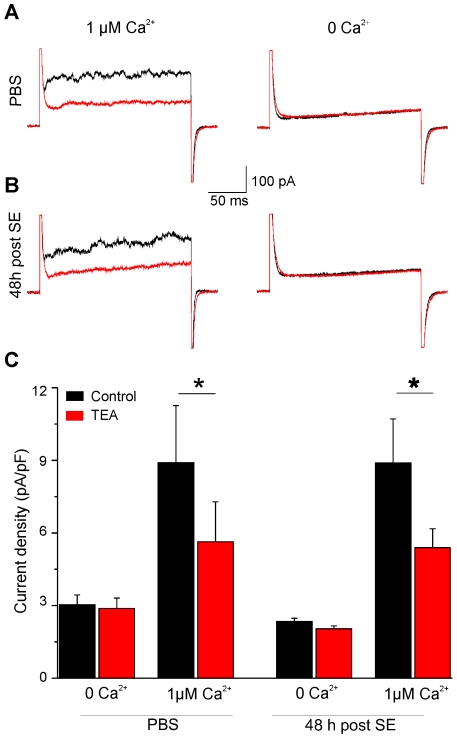
Calcium-activated potassium currents in resting and activated microglia. A, B Examples of currents induced by voltage steps from 0 to +80 mV before (black traces) and after (red traces) bath application of TEA (1 mM) with 1 µM (left panel) and 0 µM (right panel) estimated intracellular free calcium in microglial cells from control (A) and from epileptic (B) mice. C Summary of the effects of TEA (1 mM, red column) and intracellular calcium on the current densities induced by voltage steps from 0 to +80 mV in microglia of control and epileptic mice (paired *t test*, *p<0.05).

Finally, we tested whether calcium-activated potassium channels could contribute to the outward currents measured in activated microglia. Chelating intracellular calcium by including 10 mM BAPTA in the intracellular recording solution had no effect on the general aspect of the I/V curves, and no significant difference (p = 0.63, t test) was observed between outward current densities elicited by a step from −70 to +30 mV (n = 5) compared to control experiments (n = 5, data not shown). However, the recording conditions used to study Kdr currents may not favour the detection of calcium-activated potassium currents. We therefore adopted the recordings conditions (see material and methods) used previously by others [14], [26] to test directly whether calcium-activated potassium currents were up-regulated after SE. In the presence of 1 µM internal calcium (4.43 mM Ca^2+^, 5 mM BAPTA), hippocampal microglia from control (i.e. PBS-injected) mice held at 0 mV and stepped at +80 mV displayed an outward current which was reversibly inhibited by 1 mM TEA (Fig. 6A, C). Consistent with the involvement of calcium-activated potassium channels, this TEA-sensitive current was not observed in the absence of intracellular calcium (no added calcium and 5 mM BAPTA; Fig. 6A, C). Similar results were obtained with activated microglia 48 h after SE (Fig. 6B, C) and no difference was observed in the TEA-sensitive current densities between resting and activated microglia (p>0.5). These results therefore confirm the expression of calcium-activated potassium channels by microglia but also indicate that the current mediated by these channels is not up-regulated after SE.

## Discussion

The purpose of the present study was to identify which channels mediate the outward currents of hippocampal microglia activated after SE. We found no sign for the up-regulation of calcium-activated potassium currents after SE. However, biophysical and pharmacological analyses favour the involvement of potassium channels of the Kdr family containing Kv1.3 subunits.

### Comparison with cloned Kv subunits

Among the several subunits of the Kv family which are expressed by microglia in different models of activation, Kv1.1, Kv1.2, Kv1.3, Kv1.5, Kv1.6 and Kv3.1 could in theory mediate Kdr currents in hippocampal microglia (see introduction for references). However, comparison of the pharmacological and biophysical properties of the currents generated by these subunits in expression systems with those of Kdr current of activated hippocampal microglia argues against a predominant contribution of of several of these subunits in microglia. The relatively small effect of α-dendrotoxin excludes a major contribution of Kv1.1, Kv1.2 and Kv1.6 [24], [27]–[29]. The fact that 1 mM TEA had only a minor inhibitory effect on these currents also argues against a major role of Kv1.1 and Kv3.1 for which IC 50 values of TEA are 3 to 10 times lower than this concentration (table 1; [24], [28]). The observation that TEA, even at 5 mM, did not shift the activation and inactivation curves of the current is an additional evidence against the involvement of Kv3.1 which activates and inactivates at more depolarized potentials than those measured in microglia (table 1; [24], [30], [31]).

Thus, the two most likely candidates responsible of Kdr currents in hippocampal microglia are Kv1.3 and Kv1.5. The absence of Kv1.5 subunit selective inhibitors did not allow probing directly for the functional expression of this subunit in activated microglia. Yet, the biophysical properties of Kdr currents of hippocampal microglia seem to exclude a predominance of homomeric Kv1.5 channels. Indeed, the potential for half-maximal activation of Kv1.5 channels is usually more depolarized than that observed in hippocampal microglia (see table 1; [24], [30], [32]–[35]. In addition, Vicente *et al*. (2006) showed that homomeric Kv1.5 channels expressed in HEK cells show little steady-state inactivation, which is in marked contrast with the profound inactivation we observed in hippocampal microglial cells. Interestingly, the same study showed that homomeric Kv1.3 and heteromeric Kv1.3/Kv.1.5 channels do inactivate [25]. Accordingly, we found that Kdr currents were inhibited by two toxins known to block Kv1.3 containing channels, AgTx and margatoxin [36], [37]. The fact that the latter one inhibited half of the current at 1 nM and almost abolished it at 10 nM favours the conclusion that microglia Kdr currents are supported by Kv1.3 homomers rather than by Kv1.3/Kv1.5 heteromers, and excludes the presence of Kv1.5 homomeric channels, the inhibition of which requires more than 10 times higher concentrations of margatoxin [25].

### Comparison with microglia/macrophages in culture

Several biophysical properties of the Kdr currents found in hippocampal microglia after SE resemble those recorded in microglia obtained from tissue print and maintained in culture for more than a week. In these conditions, microglial cells express predominantly Kv1.3 subunits and their Kdr currents are characterized by a potential for half-maximal activation of −27 mV and a potential for steady-state half inactivation of −38 mV [15] which compare favourably with those of activated hippocampal microglia (−24.05±0.53 mV and −35.22±0.53 mV, respectively). Although not identical, the time constants for inactivation measured during long duration depolarizing pulses were also comparable in both cases (400–600 ms in cultured microglia and 290±18 ms in our study). These properties differ markedly from those reported by the same authors for the Kdr currents of microglia at earlier time points in culture and which are dominated by Kv1.5 subunits [15]. Indeed, Kdr currents of cultured microglia with a predominant expression of Kv1.5 activate at more depolarized potentials (V_1/2_ for activation near 10 mV) and inactivate with a slower time constant (1200 ms; [15]. However, two characteristics of the Kdr currents of hippocampal microglia do not fit with those of Kv1.3-expressing cultured microglia. The first one concerns agitoxin-2 for which, compared to Kv1.3-expressing microglia [15], a ten times higher concentration was needed to block Kdr currents of hippocampal microglia in acute slices. Apart from a problem of penetration of this toxin in the slice, there is no clear explanation for this difference. Yet, it is worth noting that even in culture, the concentration used to block Kv1.3-mediated currents in microglia is much higher than that used on cloned subunits in heterologous expression systems [15], [36]. The second difference concerns the cumulative inactivation. Kdr currents evoked in Kv1.3-expressing cultured microglia were characterized by a marked cumulative inactivation which leads to more than 50% of current inhibition with inter-pulse intervals of 10 sec [15]. In contrast, a similar amount of inactivation was achieved in activated hippocampal microglia only with inter-pulse intervals smaller than 0.5 sec (see Fig. 1C2). Interestingly, the presence of Kv1.5 together with Kv1.3 in heteromeric combinations seems to reduce its amplitude in macrophages and in LPS treated cultured microglia [13], [22]. Yet, the expression of Kv1.3/Kv1.5 heteromers in hippocampal microglial cells does not fit well with the sensitivity to margatoxin, the activation curves and the steady-state inactivation of Kdr currents in these cells. Altogether, these observations therefore suggest that Kv1.3 is the predominant Kv subunit expressed by activated microglia in the epileptic hippocampus. Whether a low proportion of Kv1.5 contributing to heteromeric combinations or the existence of post-transcriptional mechanisms determines some of the functional properties of these Kdr channels is however difficult to test in the absence of specific inhibitors of Kv1.5 subunits.

### Possible roles of Kv1.3 subunits in activated microglia after SE

The expression of potassium channel subunits mediating Kdr currents in activated microglia seems to control several functional parameters of the activation process. Kv1.3, but also Kv1.5, subunits expressed by activated microglial cells modulate their proliferation [13], [15]. The presence of Kv1.3-containing channels in activated microglia is therefore in keeping with our previous observation that the number of microglial cells increased in the hippocampus after SE and that many of these cells also expressed markers of proliferation such as Ki67 and MAC2 [10]. However, the exact role of these subunits in microglia proliferation remains controversial. Pannasch and collaborators reported that up-regulation of Kv1.3 and of Kv1.5 rather decreases microglia proliferation induced by LPS in culture or by nerve lesion *in vivo* [13]. On the contrary, Kotecha and Schilchter (1999) observed that blocking either of these subunits decreases the proliferation rate of un-stimulated microglia in long term cultures. The reason for such a difference is not clear but may rely on the different types of stimulation leading to microglia activation. Thus, in the case of microglia activated by SE, it would be interesting to clarify whether the up-regulation of Kv1.3 subunits promotes or inhibits their proliferation.

Finally, Schlichter and collaborators have clearly established that Kv1.3 subunit blockers reduce the NADPH-mediated respiratory burst of activated microglia and therefore the subsequent production of superoxide and free radicals which contributes to the deleterious effect of these cells on neuronal survival [16]. The inflammatory reaction which occurs in the hippocampus after SE is accompanied by neuronal cell death, even in models in which the susceptibility to seizures is the lowest [10], [38]. Whether this neuronal death is a consequence of microglia activation and of the up-regulation of Kv1.3 in these cells is an appealing hypothesis which remains to be tested.
